# Clinicians’ Perspectives on Proactive Patient Safety Behaviors in the Perioperative Environment

**DOI:** 10.1001/jamanetworkopen.2023.7621

**Published:** 2023-04-11

**Authors:** Caoimhe Duffy, Neil Menon, David Horak, Geoffrey D. Bass, Ruchika Talwar, Cara Lorenzi, Christina Taing Vo, Chienhui Chiang, Justin B. Ziemba

**Affiliations:** 1 Department of Anesthesiology & Critical Care, Perelman School of Medicine at the University of Pennsylvania, Philadelphia; 2 Department of Perioperative & Procedural Services, Hospital of the University of Pennsylvania, Philadelphia, PA; 3 Division of Urology, Department of Surgery, Hospital of the University of Pennsylvania, Perelman School of Medicine, University of Pennsylvania, Philadelphia, Pennsylvania; 4 Department of Biological Sciences, University of Delaware, Newark; 5 Department of Medicine, Perelman School of Medicine at the University of Pennsylvania, Philadelphia; 6 Division of Pulmonary, Allergy, and Critical Care Medicine, Perelman School of Medicine at the University of Pennsylvania, Philadelphia; 7 Division of Transplant Surgery, Department of Surgery, Hospital of the University of Pennsylvania, Philadelphia; 8 Department of Clinical Effectiveness and Quality Improvement, Hospital of the University of Pennsylvania, Philadelphia

## Abstract

**Importance:**

The perioperative environment is hazardous, but patients remain safe with a successful outcome during their care due to staff adaptability and resiliency. The behaviors that support this adaptability and resilience have yet to be defined or analyzed. One Safe Act (OSA), a tool and activity developed to capture self-reported proactive safety behaviors that staff use in their daily practice to promote individual and team-based safe patient care, may allow for improved definition and analysis of these behaviors.

**Objective:**

To thematically analyze staff behaviors using OSA to understand what may serve as the basis for proactive safety in the perioperative environment.

**Design, Setting, and Participants:**

This qualitative thematic analysis included a convenience sample of perioperative staff at a single-center, tertiary care academic medical center who participated in an OSA activity during a 6-month period in 2021. All perioperative staff were eligible for inclusion. A combined deductive approach, based on a human factor analysis and classification framework, as well as an inductive approach was used to develop themes and analyze the self-reported staff safety behaviors.

**Exposures:**

Those selected to participate were asked to join an OSA activity, which was conducted in-person by a facilitator. Participants were to self-reflect about their OSA (proactive safety behavior) and record their experience as free text in an online survey tool.

**Main Outcome and Measures:**

The primary outcome was the development and application of a set of themes to describe proactive safety behaviors in the perioperative environment.

**Results:**

A total of 140 participants (33 nurses [23.6%] and 18 trainee physicians [12.9%]), which represented 21.3% of the 657 total perioperative department full-time staff, described 147 behaviors. A total of 8 non–mutually exclusive themes emerged with the following categories and frequency of behaviors: (1) routine-based adaptations (46 responses [31%]); (2) resource availability and assessment adaptations (31 responses [21%]); (3) communication and coordination adaptation (23 responses [16%]); (4) environmental ergonomics adaptation (17 responses [12%]); (5) situational awareness adaptation (12 responses [8%]); (6) personal or team readiness adaptation (8 responses [5%]); (7) education adaptation (5 responses [3%]); and (8) social awareness adaptation (5 responses [3%]).

**Conclusions and Relevance:**

The OSA activity elicited and captured proactive safety behaviors performed by staff. A set of behavioral themes were identified that may serve as the basis for individual practices of resilience and adaptability that promote patient safety.

## Key Points

**Question:**

What are the behavioral categories that support perioperative staff adaptability and resilience to deliver individual and team-based safe care in the perioperative environment?

**Findings:**

This qualitative thematic analysis of self-reported proactive safety behaviors included a convenience sample of perioperative staff from a tertiary care academic medical center who participated in a facilitated activity during a 6-month period in 2021. A total of 147 unique behaviors were identified, which were categorized into 8 non–mutually exclusive themes.

**Meaning:**

The set of behavioral themes identified in this analysis may serve as the basis for individual and team-based practices of resilience and adaptability that promote patient safety.

## Teaser

This qualitative study uses thematic analysis to describe clinician perspectives on proactive safety behaviors in the perioperative environment at a US tertiary care center.

## Introduction

The perioperative environment is complex, dynamic, and error-prone, with patients more likely to experience preventable harm during perioperative care than any other type of health care encounter.^1^ Despite this, virtually all surgical cases are performed safely and effectively, demonstrating the resilience of individuals and surgical teams.^2^

Smith and Plunkett^3^ have described resilience as the “positive adaptability within systems that allows good outcomes in the presence of both favorable and adverse conditions.” Understanding this proactive adaptability and variability is the foundation of the health care Safety-II framework.^4^ In Safety-II, the focus is on how work is done at the frontline by health care professionals in real work conditions (what we refer to as *work as done*) to generate acceptable, safe outcomes almost universally (*what goes right*). This is in contrast to the traditional approach to safety management^3^ that relies on a conceptualized (*work as imagined*) model of how work should be performed for later comparison after an adverse event (*what went wrong*).

Assessing what goes right begins by connecting with frontline health care professionals because these are the individuals who understand the unique demands, concerns, and risks in their clinical areas. Their experience gives them rich insight and solutions across a range of ever-evolving clinical and organizational situations to which they continually adapt to deliver optimal care. These insights are essential to enhance safe care further and grow a robust safety culture within an institution, and to counteract the fact that organizations struggle to gather and learn and promote these experiences.^5^

Although the Safety-II framework holds promise to strengthen patient safety, it has yet to be widely adopted and inculcated among health care professionals and staff, particularly in the perioperative environment. Available tools such as the Perioperative Staff Safety Assessment^6^ from the Agency of Healthcare Research and Quality (AHRQ) and, more recently, the Bedside Learning Coordinator (BLC)^5^ from the National Health Service (NHS) rely on the traditional approach, focusing on what went wrong rather than what goes right.^5,7^

Therefore, we developed a simple, efficient, and effective tool, termed One Safe Act (OSA), capable of capturing, cataloging, and highlighting proactive safety behaviors and actions that staff of any role utilize in their daily practice to promote individual and team-based safe patient care. We hypothesized that this activity would contribute to situated learning among staff whereby they gain comfort identifying and acknowledging what goes right in their clinical work environment through socialization, participation, and collaboration with their colleagues to reinforce safety culture. These staff adaptions and behaviors can then be analyzed to identify common themes that may serve as the basis for proactive safety in a clinical unit.

## Methods

This study was approved by the University of Pennsylvania institutional review board with informed consent waived as this was deemed to be a quality improvement study. The Standards for Reporting Qualitative Research (SRQR) reporting guideline were followed for transparency.

### Theoretical Framework

Situated learning theory surmises that individual learning occurs within a sociocultural context through legitimate peripheral participation of activities within a community of practice.^8^ In this scenario, novice members begin learning how to behave, act, and identify through social interactions with more senior members around activities customary or connected to the practices of the community.^8^ This learning is context specific, informal, experiential, participatory, and opportunistic.^8^ The perioperative environment, particularly an individual operating room, is a unique example of a small but robust and co-located community of practice where members share similar behaviors, language, experiences, identities, and practices. It is these characteristics that make the perioperative environment ripe for learning and growth of health professionals through activities that influence the sociocultural norms of the community, such as with the OSA activity.

### Activity Design

**Figure 1.**
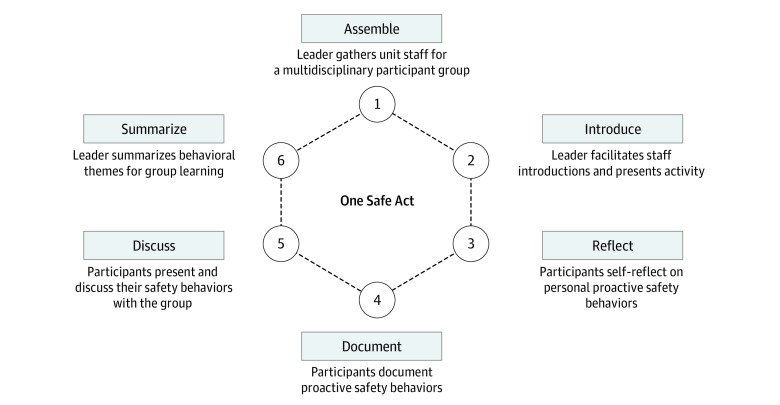
One Safe Act Activity Diagram

Briefly, in the OSA activity, a local clinical leader serves as the facilitator, gathering an ad hoc group of multidisciplinary and co-located health professionals (Figure 1). The facilitator provides an example of their own “one safe act,” defined as an action or behavior that they utilize in their daily practice to promote individual and team-based safe patient care. A pause occurs for participants to self-reflect on their own one safe act. Participants record these actions and behaviors in an online survey tool via their mobile device for cataloging and later thematic analysis. Participants are then required to share their one safe act to reinforce socialization, and build shared, potentially new knowledge and community practices focused on proactive safety behaviors. The facilitator concludes the activity with a thematic summary of the presented one safe acts.

The activity is versatile and can be performed rapidly by any staff member on any clinical unit at any time. It requires no preparation work or supplies other than a participant’s mobile device. It has no minimum number or limit on participants. It is designed to be performed within the work environment during a natural pause in daily events. It can be performed only once or repeatedly, either as a standalone activity or as part of a larger safety program. It has no risk management concerns as it is not associated with discussing or disclosing adverse events. Documented proactive safety behaviors can be immediately disseminated via discussion during the activity or provide institutional learning later via thematic analysis.

### Setting and Context

This study was conducted within the Perioperative Department of the Hospital of the University of Pennsylvania from December 2020 to July 2021. The Perioperative Department is an interprofessional and interdisciplinary functional unit responsible for managing the daily operations of the procedural areas, including the perioperative care units, operating rooms, endoscopy suites, and interventional cardiology suites.

### Study Population

All 657 perioperative staff were eligible for inclusion. This included such roles as preoperative, operative, and recovery nurse; nurse anesthetist; certified nursing assistant; radiology technicians; environmental services technicians; instrument storage and processing technicians; and administrative staff. In addition, all faculty, fellows, and residents from the surgical subspecialties and anesthesiologists were also available for inclusion. When an activity facilitator was available on the unit, a convenience sample of staff were selected for participation from among those randomly assigned to work that day in support of operating room functions. Staff were potentially able to participate more than once. The activity was conducted during a natural pause in the workflow, such as when equipment was ready, but staff were awaiting patient transport to the room.

### Data Collection

Free-text narrative descriptions of the behaviors or actions that promote individual and team-based safe patient care were self-reported by each participant in an online survey tool (eFigure in Supplement 1). All responses were captured anonymously.

### Data Analysis

Free-text narrative responses were examined using thematic analysis.^9^ Initially, these responses were examined to determine if each participant had described only 1 or more than 1 behavior. If more than 1 behavior was described, then each was separated out for individual analysis. Next, a set of preliminary codes was developed by the study author (C.D.) based on the human factor analysis and classification framework.^10^ Each of these codes were then independently applied to the entire data set by 4 study coauthors (C.D., J.Z., N.M., and D.H.). Initially, a total of 3 potential codes were allowed to be applied to each narrative response to ensure the full breadth of behaviors were identified. The group then met to review the primary, and if applicable, the secondary and tertiary codes applied to each narrative response by each author. During this session, the codes were refined based on group consensus and informed by the descriptions themselves via an inductive approach. This process was then repeated in an iterative fashion for 6 additional rounds until saturation occurred and stable themes emerged. These themes were informed by the data, but also by the authors training and experience.

## Results

Of 657 perioperative department full-time staff who were eligible, 140 participants (21.3%) described 147 behaviors during the study period. Nearly a quarter (33 participants [23.1%]) were operating room nurses (Table 1).

**Table 1.** Characteristics of Participants

Role	Participants, No. (%) (N = 140)
Nursing, operating room	33 (23.6)
Physician, trainee	18 (12.9)
Other	13 (9.3)
Nursing, prep	12 (8.6)
Nursing, recovery	11 (7.9)
Physician, faculty	9 (6.4)
Surgical technician	7 (5.0)
Radiology technician	5 (3.6)
Surgical services assistant	5 (3.6)
Transport	5 (3.6)
Certified nurse anesthetist	5 (3.6)
Medical student	3 (2.1)
Administration/leadership	3 (2.1)
Unit clerk	3 (2.1)
Perfusion technician	2 (1.4)
Facilitator	2 (1.4)
Service partner	2 (1.4)
CRNA	1 (0.7)
Environmental services	1 (0.7)
Anesthesia technician	0
Instrument processing	0
Pharmacist	0
Biomedical/clinical engineering	0

Abbreviation: CRNA, certified registered nurse anesthetist.

A total of 8 non–mutually exclusive themes emerged (Table 2). Briefly, these included (1) routine-based adaptation, defined as ensuring that a routine, repetitive, highly practiced task relating to procedure, training, or proficiency is completed correctly; (2) resource availability and assessment adaptation, defined as reviewing and confirming that all required resources are present and functioning; (3) education adaptation, defined as providing staff with new information that prepares them with the knowledge to perform a task in a standard or safe way; (4) environmental ergonomics adaptation, defined as ensuring that the environment and equipment within it are configured appropriately for user interaction; (5) situational awareness adaptation, defined as assessing situations for potential errors that could arise, with adjustments to behavior to defend against these errors from occurring; (6) communication and coordination adaptation, defined as the use of communication, coordination, teamwork, and planning to prevent errors and enhance safety; (7) personal or team readiness adaptation, defined as pre-duty or on-duty activities or standard preparations required to perform optimally; and (8) social awareness adaptation, defined as attending to the social and emotional needs of others, including patients and staff. These themes were applied via consensus by the author coding group to each narrative description for categorization. A primary theme was applied to each with a secondary theme applied when necessary.

**Table 2.** Proactive Safety Behavior Thematic Classification Scheme

Name	Description
Routine-based adaptation	Ensuring a routine, repetitive, highly practiced task relating to procedure, training, or proficiency is completed correctly. Failure to do this task (eg, checklist completion, checking allergies, using a certain piece of equipment every time a specific process is performed) could result in an unsafe situation
Resource availability and assessment adaptation	Reviewing and confirming that all required resources (including equipment, controls, and staffing) are present and functioning correctly (eg, checking that an ultrasound machine is available, ensuring that additional team members are available to assist). This can be an ongoing process
Education adaptation	Providing staff with new information that provides them with the knowledge to perform a task in a standard or safe way (eg, new defibrillator training)
Environmental ergonomics adaptation	Ensuring that the environment and equipment within it are configured appropriately for user interaction (eg, placing mats on floor to cover cable, ensuring that machines are adjusted to the correct settings). This can be an ongoing process
Situational awareness adaptation	Assessing situations for potential errors that could arise, adjusting behavior to defend against potential errors from occurring (eg, holding the door open for staff pushing a stretcher, recognizing a risk posed by beds being left unlocked, ensuring no equipment is hanging off the side of the bed while in transport, ensuring anesthetized patient safety)
Communication and coordination adaptation	Refers to the use of communication, coordination, teamwork, and planning to prevent errors and enhance safety. This includes provider-provider interactions (eg, using closed loop communication, practicing read-back, calling for help, assessing staff mix when assigning roles) and patient-provider interactions (eg, confirming laterality or procedure to be performed)
Personal or team readiness adaptation	Refers to pre-duty or on-duty activities or standard preparations required to perform optimally on the job such as adequate sleep (eg, review of appropriate medical/technical knowledge or patient-specific knowledge like chart or imaging review)
Social awareness adaptation	Attending to the social and emotional needs of others, including patients and junior colleagues (eg, putting patients at ease by showing empathy, helping patients feel more comfortable by offering to answer their questions)

**Figure 2.**
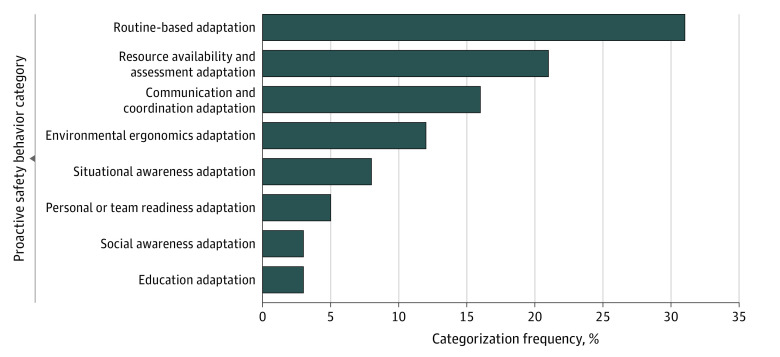
Frequency of Proactive Safety Behavioral Categories in Primary Assigned Theme

**Figure 3.**
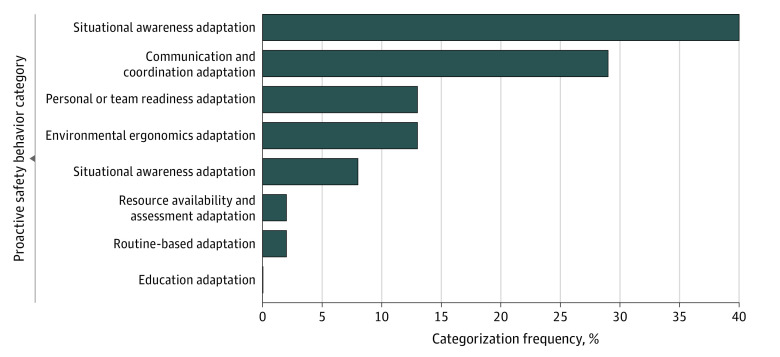
Frequency of Proactive Safety Behavioral Categories in Secondary Assigned Theme

Routine-based adaptations were the most common behaviors at 31% (46 responses) (Figure 2; Figure 3). For example, a recovery nurse wrote that they “always check Micromedex for compatibility when giving unfamiliar or infrequently used IV [intravenous] meds” and a surgical technician commented, “Making sure the patient belt is on the patient once the patient gets on the bed.” Resource availability and assessment adaptations accounted for the next most frequent behaviors at 21% (31 responses). These behaviors included examples like “wrapping transport pulse oximeter to supplemental nasal cannula used for transport” as described by a trainee physician, or “checking all the monitors in my recovery pod to make sure all alarms are on and the monitors are working before the start of my shift,” as suggested by a recovery nurse. Communication and coordination adaptation represented 16% of the behaviors (23 responses). These behaviors included such actions as “always ask patients when checking in what side we are doing to verify they understand and are comfortable and I myself know […] then together we mark the correct side,” which was described by a faculty physician, or, “I make sure I know who the covering provider is and where to find the phone numbers,” which was submitted by a recovery nurse. Environmental ergonomics adaptation accounted for the final large category of behaviors with 12% (17 responses). This category consisted of behaviors such as “make sure all my lines (IV lines, monitor cords, O_2_, chest tubing) are clear before moving the patient to and from the stretcher and or throughout the halls,” as said by a transport technician.

The remaining 4 themes each contained a relatively small number of responses, yet still highlighted important behaviors. For example, situational awareness adaptation included “checking on patient when alarms are going off even when they’re not my own,” as said by a recovery nurse; or personal or team readiness adaptation, such as “during patient check in, pulling up and reviewing relevant imaging prior to skin marking,” as described by a trainee physician; or education adaptation like “schedule education of new equipment for the nursing staff,” from an operating room nurse; or, finally, social awareness adaptation like “asking patients if they have any questions or concerns before the procedure,” also from an operating room nurse. Additional examples of proactive safety behaviors with their associated categorization are available in eTable in Supplement 1.

## Discussion

The OSA activity was participatory and collaborative by design to reinforce the sociocultural nature of situated learning and to build shared, potentially new knowledge and community practices focused on proactive safety behaviors within the perioperative environment. The OSA activity achieved this goal by eliciting and capturing these behaviors used by perioperative team members in their daily practice to ensure or promote patient safety through group dialogue and discussion. Thematic analysis revealed that the majority of staff report proactive safety behaviors that focus on either routine-based tasks or ensuring appropriate resource availability. However, reported behaviors were still quite varied, also covering dimensions such as communication, ergonomics, and situational awareness. Individual reported behaviors were observed to be frequently cross-functional and applicable to more than a single staff role.

Patient safety should be characterized not only by the absence of accidents but also by the frequency with which intended outcomes are achieved.^11^ OSA enhances the ability of participants and leadership to recognize positive, proactive safety initiatives already present but previously unnoticed. Qualitative insights from frontline staff are seldom part of routine data capture. Failure to capture frontline knowledge and then enact local operational change contributes to health care’s slow pace of innovation adoption^12^ and can also lead to staff dissatisfaction and disengagement.^5^ OSA uniquely enables access to precious frontline knowledge, gathering data on work as done while simultaneously engaging with the larger perioperative community to begin the transformation into the practice of focusing on what goes right (ie, Safety-II) to build even greater resilience. It provides frontline staff with a psychological safe environment to engage with members of the community of practice beyond just those with a similar role (ie, staff working in anesthesia talking only with anesthesia colleagues), which is too often the case in a clinical work unit. OSA is a starting point that enables staff to focus on interdependence, creating commonality between groups, as well as an appreciation and awareness of the teamwork that occurs every day. Ideally, it is just one activity that can be continuously utilized within the context of a larger safety education and practice program at the unit, department, or institutional level to highlight and reinforce resilience within perioperative health care teams. Specifically, collected and categorized behaviors can be further strengthened in high-performing units or disseminated for learning and application in low-performing units.

The identified themes of staff adaptations around routines, resource availability and assessments, education, environmental ergonomics, situational awareness, communication and coordination, personal or team readiness, and social awareness represent a new method of categorizing proactive safety behaviors by perioperative health care professionals. These themes are largely congruent with the only other prior analysis of operating room staff assessing safety through a work as done lens.^13^ In this qualitative interview analysis of 17 staff consisting of only operating room nurses, registered nurse anesthetists, and surgeons, Göras^13^ identified only 3 universal categories: preconditions and resources, planning and preparing for the expected and unexpected, and adapting to the unexpected. Additional universal subcategories were identified—including coordinating and reaffirming information, creating a plan for the patient and undergoing mental preparation, and prioritizing and solving upcoming problems—but the remainder were occupation specific.^13^ Nevertheless, there are several similarities to our responses and themes. For example, the preconditions and resources category is equivalent to our resource availability and assessments adaption theme and the coordinating and reaffirming information category is congruent with our communication and coordination theme. Although further evaluation is necessary across other populations and settings, this suggests that a core, universal set of actions may underlie safe perioperative care.

Assessing how work is done is a relatively new lens with which to understand operating room safety. Traditionally, safety improvement processes have relied on a deficit-based model, which focused on engineering environments after an event to reduce or eliminate harm.^14^ However, this focuses only on the very small fraction of care that results in harm. It misses understanding and learning from the conditions that lead to success.^14^ Instead, utilizing a resilience engineering model, which hypothesizes that acceptable outcomes can be maintained under expected and unexpected conditions alike (ie, resilience), it is possible to focus on the system as a whole, learning from the failures, everyday successes, and exceptional successes.^14^

Previous attempts to capture this information in the operating room have occurred, most notably with black box technology.^15,16^ In a prior analysis of a single operating room with this equipment installed, general laparoscopic surgery of only 4 surgeons was assessed to capture potential resilient, proactive safety behaviors.^15,16^ In a given surgical case, a median of 12 resilient supports were identified per case, most commonly in the person or organization category.^15^ Although this begins to provide insight into resilient, proactive safety behaviors in the operating room, it focuses only on the surgical case itself, is limited to the staff in the room, and is technically challenging to implement. However, the Resilience Engineering Tool to Improve Patient Safety (RETIPS), which is an incident reporting structure to elicit narrative descriptions of clinical scenarios where safe, successful care was present to capture positive adaptations, is a more flexible approach.^17^ In the evaluation of the tool, a total of 9 anesthesia residents provided narrative examples, which were later thematically evaluated. The key themes identified in the recalled scenarios were variability in routines, preparedness, communication and coordination, and safety-efficiency relationships.^17^

However, the OSA activity can deliver similar assessments across perioperative clinical (and nonclinical) areas without the need for specialized equipment. Furthermore, our analysis is unique in that it relied on open-ended and self-reported concrete, specific actions solicited from those in the midst of active, presumably safe clinical care. This allows for a real-time, leading indicator of resilience and eliminates the potential for recall bias, such as with the RETIPS narrative submissions,^17^ or the hypothetical bias that can occur with generic scenarios about safety care planning or dealing with uncertainty. In addition, unlike traditional studies, the OSA activity can continuously provide updated proactive safety behavior analysis through repeated administration for ongoing refinement of these thematic categories and subsequent learning.

Finally, unlike black boxes or the RETIPS tool, which are individualistic reviews, OSA provides an opportunity to introduce positivity into the perioperative environment as it focuses on constructive actions and behaviors within a community of practice. Focusing on these proactive actions can aid engagement, pleasure, and a sense of meaning in the workplace for staff, which are all linked to positive organizational outcome.^18^ OSA allows staff to foster positive emotions and participate in collective reflection, which are recognized methods in assisting individuals develop resilience.^19^ Individuals who cultivate positive factors can use them to cope with negative emotions.^18^ An analogy between OSA can be drawn with Three Good Things, an intervention used as an intentional activity to cultivate positive cognitions and emotions.^20^ Similarly, further studies could focus on whether OSA has similar long-term positive psychological outcomes, particularly on reduction of staff burnout, and through thematic analysis of the narrative responses, which can unlock the behaviors that support the capability of health care teams to almost universally deliver safe care.

### Limitations

This study had several limitations. First, this was a programmatic evaluation of OSA that did not measure the acquisition of new proactive safety behaviors or changes in clinical practice to facilitate safe care. There was no correlation of patient or clinical outcomes as a result of participation in OSA. This was by design, as focusing on proactive safety behaviors to build system resilience rather than the traditional approach of retrospective analysis of a safety event to initiate change is a major paradigm shift in how health care professionals think and act with regards to patient safety. Therefore, at this stage, the objective is to increase awareness, comfort, and acceptance of focusing on proactive safety behaviors among staff within the clinical environment, which is why currently practiced behaviors were selected as a primary outcome with a focus on understanding what actions may support safe care. Future iterations of this activity will allow for the assessment of new or changed proactive safety behaviors as a result of participation, and application of these behavioral themes to self-reported or observed behaviors of health care staff in other clinical environments will be necessary to validate these findings.

## Conclusion

The OSA activity elicited and captured proactive safety behaviors performed by staff within the perioperative environment through a participatory and collaborative design to reinforce the sociocultural nature of situated learning, and build shared, potentially new knowledge and community practices to promote safety in their work setting. The key benefits of this approach are an opportunity to highlight and understand behaviors that align with the Safety-II principle to focus on what goes right in perioperative settings. This has revealed a set of behavioral themes that may serve as the basis for individual and team-based practices of resilience and adaptability that promote patient safety.

## Notes

Supplement 1. **eFigure.** One Safe Act Activity Survey Tool **eTable.** Examples of Proactive Safety Behaviors Submitted by Participants

Supplement 2.
Data Sharing Statement
