# Supplementary material for: Clinicians’ Perspectives on Proactive Patient Safety Behaviors in the Perioperative Environment
Source: JAMA Netw Open. 2023 Apr 11;6(4):e237621. doi: 10.1001/jamanetworkopen.2023.7621 (PMC10091176; doi:10.1001/jamanetworkopen.2023.7621)
Supplement: Supplement 1. — eFigure. One Safe Act Activity Survey Tool eTable. Examples of Proactive Safety Behaviors Submitted by Participants [file jamanetwopen-e237621-s001.pdf]

Supplemental Online Content

Duffy C, Menon N, Horak D, et al. Clinicians' perspectives on proactive patient safety behaviors in the perioperative environment. *JAMA Netw Open.* 2023;6(4):e237621. doi:10.1001/jamanetworkopen.2023.7621

eFigure. One Safe Act Activity Survey Tool

eTable. Examples of Proactive Safety Behaviors Submitted by Participants

This supplemental material has been provided by the authors to give readers additional information about their work.

© 2023 Duffy C et al. *JAMA Network Open.*

eFigure. One Safe Act Activity Survey Tool

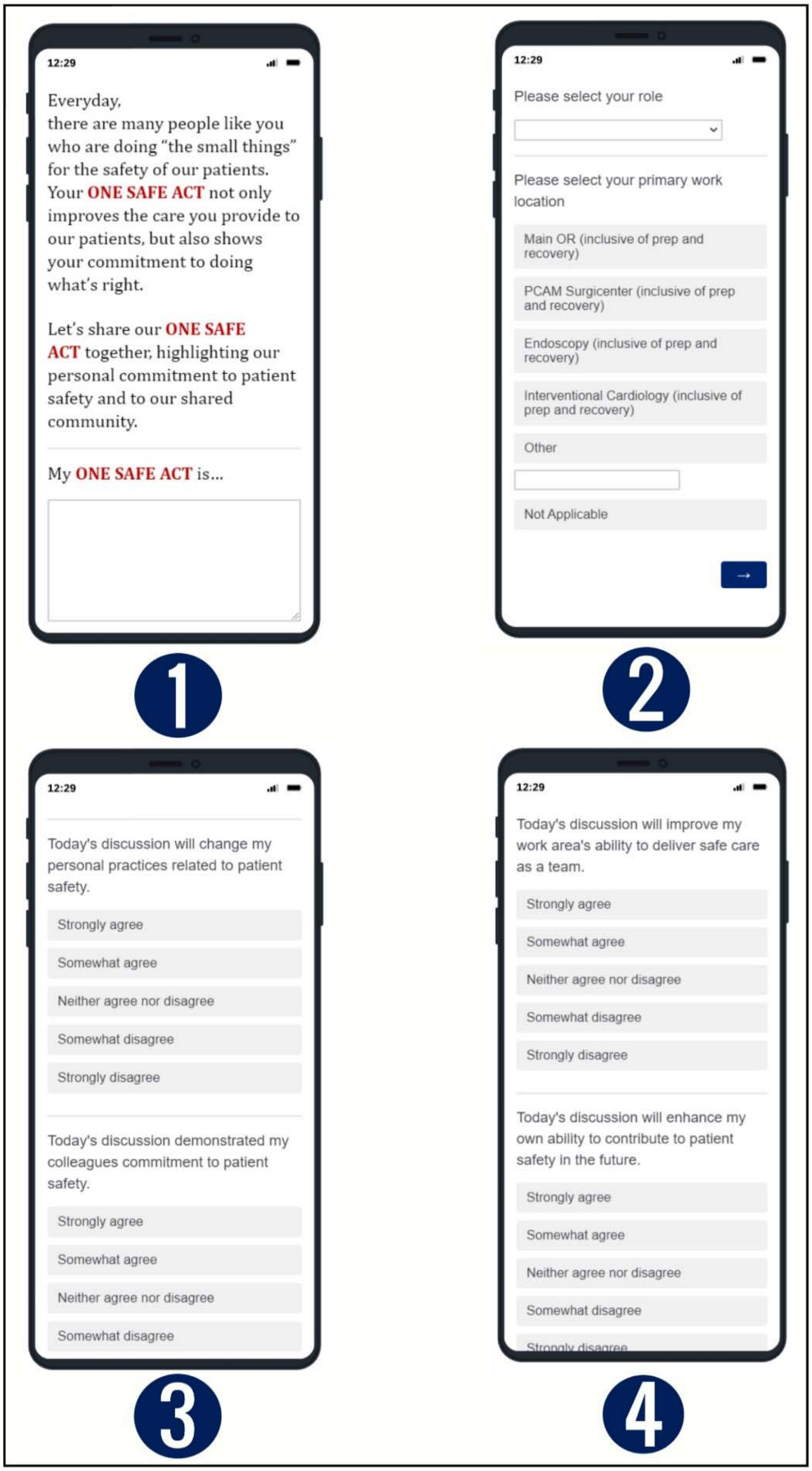

© 2023 Duffy C et al. JAMA Network Open.

eTable. Examples of Proactive Safety Behaviors Submitted by Participants

**Examples of Proactive Safety Behaviors Submitted by Participants**		
**Response**	**Role**	**Categorization**
"I always verify name and date of birth when I check in operative patients in the pre op area to be sure I am in the right chart and in the right bay with the right patient."	Faculty Physician	Routine Based Adaptation
"During patient check in, pulling up and reviewing relevant imaging prior to skin marking."	Trainee Physician	Personal or team readiness adaptation
"Always check Micromedex for compatibility when giving unfamiliar or infrequently used IV meds."	Recovery Room Nurse	Routine Based Adaptation
"Lock all the stretchers in the pre-operative area every morning to make sure when we bring the patients back they are safely able to get onto the stretcher."	Medical Assistant	Environmental ergonomics adaptation
"Ensuring employee safety in the OR by covering cords with mats, cleaning up spills immediately."	Service Partner	Environmental ergonomics adaptation
"Having an additional nurse check the setup of patient controlled analgesia and epidurals."	Recovery Room Nurse	Resource availability and assessment adaptation
"Always verify patient information in terms of allergies and ensure operative supplies are safe for listed allergies."	Surgical Technologist	Routine Based Adaptation

© 2023 Duffy C et al. JAMA Network Open.

"Checking identification bracelet to make sure their name and date of birth is correct."	Unit Secretary	Routine Based Adaptation

© 2023 Duffy C et al. JAMA Network Open.
