# Supplementary material for: Clinicians’ Perspectives on Proactive Patient Safety Behaviors in the Perioperative Environment
Source: JAMA Netw Open. 2023 Apr 11;6(4):e237621. doi: 10.1001/jamanetworkopen.2023.7621 (PMC10091176; doi:10.1001/jamanetworkopen.2023.7621)
Supplement: Supplement 2. — Data Sharing Statement [file jamanetwopen-e237621-s002.pdf]

Data Sharing Statement

Duffy. Clinicians' Perspectives on Proactive Patient Safety Behaviors in the Perioperative Environment. *JAMA Netw Open*. Published April 11, 2023.
doi:10.1001/jamanetworkopen.2023.7621

Data
Data available: Yes
Data types: Deidentified participant data
How to access data: Data can be provided after a reasonable request by contacting senior author.
When available: With publication

Supporting Documents
Document types: Statistical/analytic code
How to access documents: Data can be provided after a reasonable request by contacting senior author.
When available: With publication

Additional Information
Who can access the data: Data can be provided after a reasonable request by contacting senior author.
Types of analyses: Data can be provided after a reasonable request by contacting senior author.
Mechanisms of data availability: Data can be provided after a reasonable request by contacting senior author.
